# A Bilingual On-Premises AI Agent for Clinical Drafting: Implementation Report of Seamless Electronic Health Records Integration in the Y-KNOT Project

**DOI:** 10.2196/76848

**Published:** 2025-11-24

**Authors:** Hanjae Kim, So-Yeon Lee, Seng Chan You, Sookyung Huh, Jai-Eun Kim, Sung-Tae Kim, Dong-Ryul Ko, Ji Hoon Kim, Jae Hoon Lee, Joon Seok Lim, Moo Suk Park, Kang Young Lee

**Affiliations:** 1 Department of Biomedical Systems Informatics, College of Medicine, Yonsei University, 50-1, Yonsei-Ro, Seodaemun-gu, Seoul, 03722, Republic of Korea, 82 22282500; 2 PHI Digital Healthcare, Seoul, Republic of Korea; 3 Yonsei Institute for Digital Health, Yonsei University, Seoul, Republic of Korea; 4 Department of Medical Records, Severance Hospital, Yonsei University Health System, Seoul, Republic of Korea; 5 Saltlux Inc, Seoul, Republic of Korea; 6 Department of Emergency Medicine, College of Medicine, Yonsei University, Seoul, Republic of Korea; 7 Department of Anesthesiology and Pain Medicine, Anesthesia and Pain Research Institute, College of Medicine, Yonsei University, Seoul, Republic of Korea; 8 Department of Radiology, College of Medicine, Yonsei University, Seoul, Republic of Korea; 9 Division of Pulmonary and Critical Care Medicine, Department of Internal Medicine, College of Medicine, Yonsei University, Seoul, Republic of Korea; 10 Department of Surgery, College of Medicine, Yonsei University, Seoul, Republic of Korea

**Keywords:** artificial intelligence agent, large language models, documentation, electronic health records, insights

## Abstract

**Background:**

Large language models (LLMs) have shown promise in reducing clinical documentation burden, yet their real-world implementation remains rare. Especially in South Korea, hospitals face several unique challenges, such as strict data sovereignty requirements and operating in environments where English is not the primary language for documentation. Therefore, we initiated the Your-Knowledgeable Navigator of Treatment (Y-KNOT) project, aimed at developing an on-premises bilingual LLM-based artificial intelligence (AI) agent system integrated with electronic health records (EHRs) for automated clinical drafting.

**Objective:**

We present the Y-KNOT project and provide insights into implementing AI-assisted clinical drafting tools within constraints of health care system.

**Methods:**

This project involved multiple stakeholders and encompassed three simultaneous processes: LLM development, clinical co-development, and EHR integration. We developed a foundation LLM by pretraining Llama3-8B with Korean and English medical corpora. During the clinical co-development phase, the LLM was instruction-tuned for specific documentation tasks through iterative cycles that aligned physicians’ clinical requirements, hospital data availability, documentation standards, and technical feasibility. The EHR integration phase focused on seamless AI agent incorporation into clinical workflows, involving document standardization, trigger points definition, and user interaction optimization.

**Implementation (Results):**

The resulting system processes emergency department discharge summaries and preanesthetic assessments while maintaining existing clinical workflows. The drafting process is automatically triggered by specific events, such as scheduled batch jobs, with medical records automatically fed into the LLM as input. The agent is built on premises, locating all the architecture inside the hospital.

**Conclusions:**

The Y-KNOT project demonstrates the first seamless integration of an AI agent into an EHR system for clinical drafting. In collaboration with various clinical and administrative teams, we could promptly implement an LLM while addressing key challenges of data security, bilingual requirements, and workflow integration. Our experience highlights a practical and scalable approach to utilizing LLM-based AI agents for other health care institutions, paving the way for broader adoption of LLM-based solutions.

## Introduction

### Background

Large language models (LLMs) have recently garnered significant attention, raising expectations for their applications in health care systems, encompassing clinical care support, research, and education [1,2]. However, most research has focused on implementations in the United States, and these solutions have yet to demonstrate meaningful reductions in administrative burden, as they mainly address tasks related to medical knowledge [3].

South Korea’s health care system is renowned for its efficiency, offering low costs with high accessibility and quality. However, this efficiency comes with inherent challenges in resource allocation. Health care providers often manage substantial workloads, seeing many patients in limited time frames. This situation has been particularly exacerbated by recent mass resignation of residents, which has left tertiary hospitals facing a critical shortage of human resources [4,5]. These circumstances underscore an urgent demand for meaningful assistance from LLMs.

Clinical documentation represents a significant burden for health care providers [6,7], and there is growing optimism about LLMs’ potential to alleviate this burden [8,9]. Clinical documentation involves condensing previous records, a task that LLMs excel at [10,11]. Accordingly, several studies have explored the capabilities of proprietary LLMs in generating clinical notes such as radiology referrals [12] or discharge summaries [13,14]. However, implementing existing LLM solutions in South Korea faces several unique challenges. Korean medical regulations mandate that all medical records be stored exclusively on domestic servers or clouds [15], making it impossible to utilize foreign commercial services like ChatGPT [16]. Additionally, medical documents in Korea often exhibit mixed usage of Korean and English, requiring models capable of processing bilingual clinical notes effectively [17]. Korea’s Ministry of Food and Drug Safety does not classify artificial intelligence (AI) software for documentation as a medical device unless it involves medical judgements [18], thereby exempting the requirement for regulatory approval. Nevertheless, these challenges hinder the widespread adoption of LLMs in Korea.

Although some pilot projects have attempted incorporating LLMs within electronic health records (EHRs), full-scale integration in real clinical settings remains rare. Due to their separate interface, manually retrieving information from EHRs and typing it into LLMs may ironically be time-consuming. In the study by Goh et al [19], interaction with an LLM led to increased time in patient management reasoning. For LLMs to be continuously and effectively utilized by health care providers, connecting LLMs directly to EHRs is necessary.

To address these challenges, we initiated the Your-Knowledgeable Navigator of Treatment (Y-KNOT) project, aimed at developing a hospital-dedicated AI agent that seamlessly integrates a small, bilingual LLM with existing systems for automatic clinical drafting. This paper presents our experiences and insights from developing and implementing this solution.

### Objectives

We aim to demonstrate a practical approach to leveraging LLMs within the constraints of the health care system, potentially offering a model for similar implementations in other limited-resource settings. This paper highlights the multidisciplinary process of the Y-KNOT project, key features of the final implementation, and presents a human evaluation of its feasibility. Our experience provides valuable insights into the challenges and opportunities of integrating AI-assisted clinical drafting tools in health care settings while maintaining compliance with local regulations and addressing specific linguistic requirements.

## Methods

### Ethical Considerations

This study was reviewed and approved by the Institutional Review Board (IRB No. 4-2023-003) and the Data Review Board (DRB No. 24-01-005) of Severance Hospital. All patient data used in this study were retrieved from the hospital’s research-purpose EHR database and deidentified prior to use, waiving the need for additional informed consent.

### Project Overview

The Y-KNOT project was conducted at Severance Hospital, a tertiary hospital in Seoul, South Korea. The project was initiated in June 2024 and the first service in routine clinical practice started in November 2024. The total cost of the project, including all expenses such as equipment and labor, did not exceed US $1,500,000. The final LLM model developed in this project is jointly owned by Severance Hospital and ‘PHI Digital Healthcare Co., Ltd.’ (Seoul, South Korea).

This implementation report adheres to the iCHECK-DH (Guidelines and Checklist for the Reporting on Digital Health Implementations) reporting guidelines [20] (Checklist 1).

**Figure 1.**
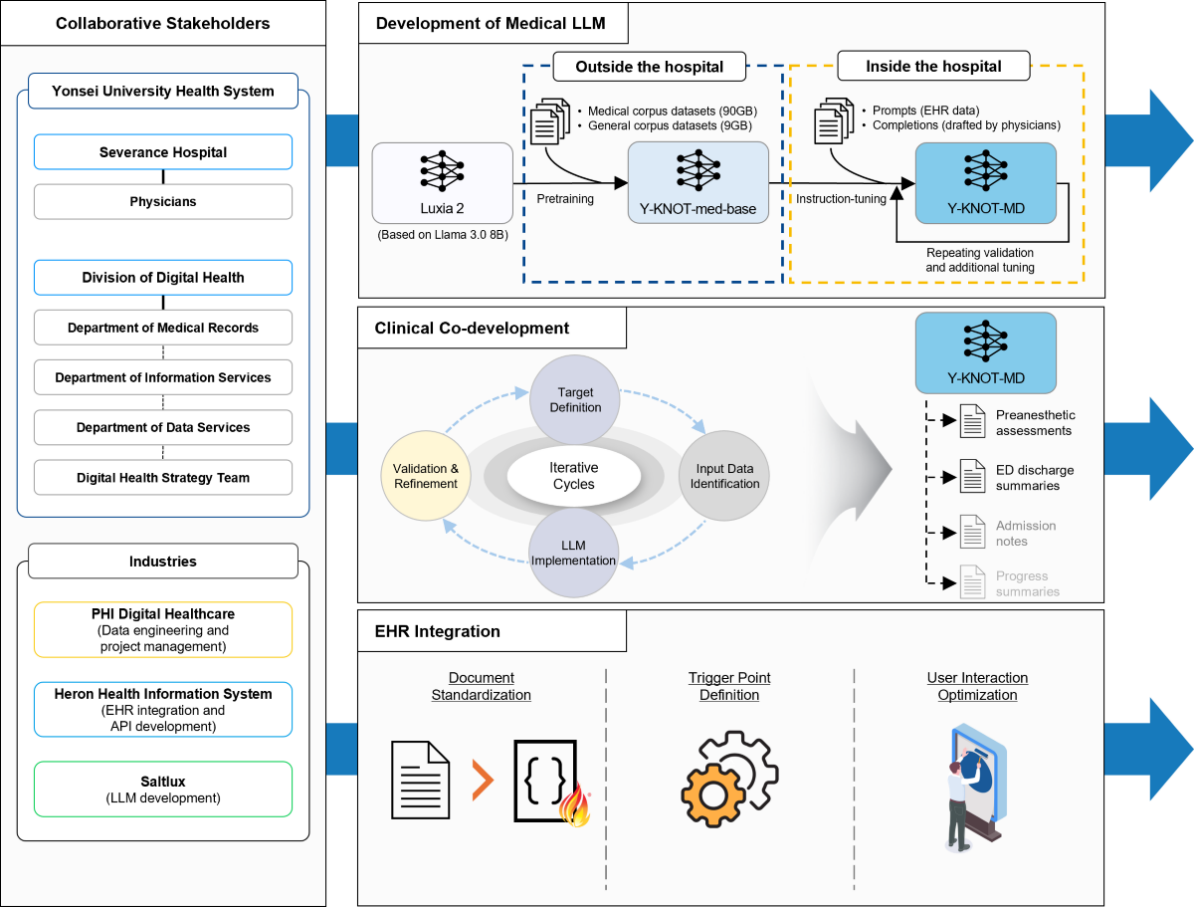
Overall landscape of the Y-KNOT project. B: billions; ED: emergency department; EHR: electronic health record; GB: gigabytes; LLM: large language model; Y-KNOT: Your-Knowledgeable Navigator of Treatment.

The project encompassed three major phases: medical foundation LLM development, clinical co-development, and EHR integration, which were carried out simultaneously. Figure 1 displays the overall project landscape.

### Development of Medical Foundation LLM

We first developed ‘Y-KNOT-med-base,’ a small, bilingual LLM for general medical purposes. We used Luxia 2 [21] developed by ‘Saltlux Inc.’ (Seoul, South Korea) as a base model, which was built upon Llama 3 (8 billion parameters) [22] and specialized for Korean language through pretraining on 1.5 terabytes of general corpus datasets. We decided to use a small model for rapid project completion, minimal latency in clinical settings, and environmental and economic sustainability. To adapt the model for medical applications, we further trained it with 90 GB of medical and 9 GB of general corpus datasets in Korean and English, consisting of open source and internally collected datasets. The pretraining data was augmented with instruction-response pairs for instruction pretraining [23], which enables better alignment with domain-specific tasks. The training was conducted outside of the hospital to ensure greater flexibility and broader reusability of the foundation model by other institutions. Hyperparameter settings are provided in Multimedia Appendix 1.

To assess its capability to understand medical knowledge, we evaluated ‘Y-KNOT-med-base’ on PubMedQA (biomedical question answering based on PubMed abstracts) [24] for English and KorMedMCQA (multichoice question answering derived from licensing examinations for doctors, nurses, and pharmacists in South Korea) [25] for Korean. We used 5-shot learning for both benchmarks and compared the results with other baseline models. Baseline models for PubMedQA were selected from the state-of-the-art models on the PubMedQA leaderboard [26] whose parameter sizes were disclosed. Baseline models and their respective results for KorMedMCQA were taken from the original KorMedMCQA paper [25], focusing specifically on nonproprietary multilingual models.

### Clinical Co-Development Phase

**Figure 2.**
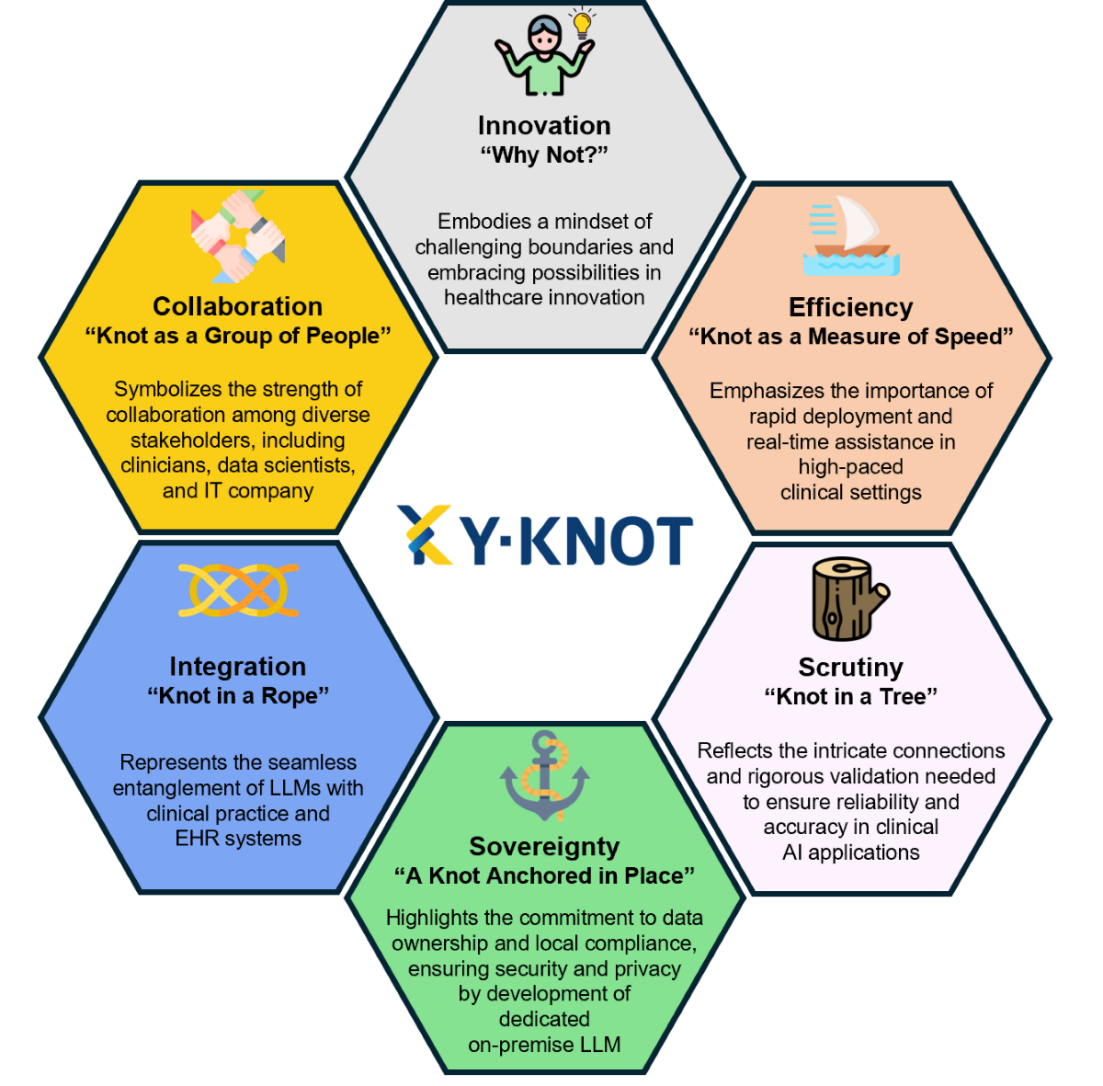
Core values of the Y-KNOT project. AI: artificial intelligence; EHR: electronic health record; IT: information technology; LLM: large language model; Y-KNOT: Your-Knowledgeable Navigator of Treatment.

The Y-KNOT project involved intensive collaboration with related departments, including physicians, data scientists, software engineers, and medical record specialists. Working closely together, we established six core values: innovation, collaboration, integration, sovereignty, scrutiny, and efficiency (Figure 2).

With the core values internalized, we conducted multiple iterative development cycles. Each cycle began with defining specific clinical documentation needs, followed by identifying available EHR data, assessing the technical feasibility of LLM implementation, and refining results through data adjustments and retraining of the model. Through these cycles, we progressively refined our understanding of automatable document types, confirmed the output templates, and determined the optimal approach for automation–whether through rule-based systems or LLM inference. This process was essential for establishing a system that not only met immediate clinical needs but also ensured standardization across departments while maintaining compliance with medical documentation requirements.

To adapt the LLM for drafting specific document types–emergency department (ED) discharge summary and preanesthetic assessment–we instruction-tuned the Y-KNOT-med-base. We called the resulting model ‘Y-KNOT-MD,’ which is an abbreviation for ‘Y-KNOT medical document’ Medical document data for the model prompts were selected from the hospital’s EHR database. Corresponding completions were prepared by physicians, addressing clinical needs while following the guidelines established by data scientists. The model was trained on 300 prompt-completion pairs for each document type. As the training involved patient data, it was conducted within the hospital environment to minimize the risk of data leakage. Details regarding hyperparameter settings are provided in Multimedia Appendix 1.

### EHR Integration Phase

In parallel with previously described phases, we conducted comprehensive EHR integration planning with the hospital’s EHR team. This phase focused on defining the optimal service architecture to seamlessly integrate the AI agent into existing clinical workflows. It encompassed three key components: medical document standardization, service trigger point definition, and user interaction optimization.

First, we screened medical document forms from the EHR system to be used for the actual service. Out of 2201 different document forms, total 989 forms were selected. The rest were excluded due to inconsistent usage, absence of textual content, or their association with surveys, referrals, palliative care or physical therapies. This decision was reached after numerous meetings with the medical records team and clinicians. Then, we standardized the selected forms based on Fast Healthcare Interoperability Resource (FHIR) [27] standards. This standardization not only enhanced interoperability for existing documentation but also established a robust framework for future development, ensuring long-term system scalability and maintainability.

Second, we mapped precise trigger points for AI agent activation to ensure assistance without disrupting existing clinical routines. The system supports both real-time triggers and batch processing. We carefully selected the optimal time for batch processing to minimize potential system load, and tested system latency to ensure that the integration would not impact the EHR’s overall performance.

Third, we established a documentation display and a user interaction framework that maximized efficiency while preserving physician control over final documentation. The interface enabled quick review and editing of AI-generated content through intuitive controls for accepting, modifying, or rejecting suggestions. This design emphasized minimal click paths to streamline the documentation process.

To ensure data sovereignty, all infrastructures including servers and databases were hosted within the hospital’s secure on-premises environment.

### Predefined Clinical Evaluation Criteria

Before the deployment in actual clinical setting, we evaluated the qualities of automatically generated ED discharge summaries and preanesthetic assessments to assess the performance of the AI agent. For each type of document, 100 pairs of input data, which had not been used during the development, and consequent model outputs were provided to 2 physicians. The physicians graded the outputs in terms of consistency, coherence, fluency, relevance, safety, subjective satisfactory rate, and usability. In addition, the impact on decision-making was graded only for preanesthetic assessments. The specific criteria for each metric are listed in Table 1. All metrics were graded using 5-point Likert Scales, except for usability, which had a maximum score of 4, and impact on decision-making, which had a maximum score of 3. Higher scores indicated better output quality for all metrics. Mean scores were calculated for all metrics, except for impact on decision-making, where the proportion for each score was calculated.

**Table 1.** Criteria for evaluating auto-generated drafts.

Metrics	Range^a^	Criteria
Consistency	1‐5	The consistency of the information provided on the output
Coherence	1‐5	The logical structure of the output in context
Fluency	1‐5	The appropriateness in grammatical, lexical, or structural aspects of the output
Relevance	1‐5	The alignment of the output with the topic
Safety	1‐5	The correctness of medical information in the output
Subjective satisfactory rate	1‐5	Subjective measurement of overall satisfaction with the output
Usability	1‐4	Whether the output can be provided to the user without modifications
Impact on decision-making^b^	1‐3	The extent to which the response influences medical judgment, categorized into three levels: positive, no impact, and negative

a A higher score indicates better quality of output in all metrics.

b This metric was used solely for evaluating preanesthetic assessments.

## Implementation (Results)

### Performance Evaluation of Medical Knowledge and Language Capabilities

The ‘Y-KNOT-med-base’ achieved an accuracy score of 75.2 on the PubMedQA. Despite its relatively small size and absence of fine-tuning process, the performance was comparable to state-of-the-art baselines which were fine-tuned on larger parameter scales. The average accuracy score was 55.8 on the KorMedMCQA (doctor: 47.0, nurse: 64.1, pharmacist: 56.2), outperforming other multilingual pretrained models on all three exam categories. Detailed performance results are provided in Tables2  3.

**Table 2.** Evaluation result of Y-KNOT-med-base^a^ on PubMedQA^b^.

Model	Accuracy
Meditron-70B	81.6
Palmyra-Med-40B	81.1
AntGLM-Med-10B	80.6
Flan-PaLM-540B	79
Y-KNOT-med-base-8B	75.2

a Y-KNOT: Your-Knowledgeable Navigator of Treatment.

b PubMedQA: PubMedQA dataset is freely available on Hugging Face[28].

**Table 3.** Evaluation result of Y-KNOT-med-base^a^ on KorMedMCQA^b^.

Model	Accuracy			
	Doctor	Nurse	Pharm	Average
Llama2-70B	42.5	63.5	53.3	53.1
Yi-34B	40	55.5	52.8	49.4
SOLAR-10.7B-v1.0	37.2	55.5	54.1	48.9
Mistral-7B-v0.1	29.8	42.1	43.5	38.5
Y-KNOT-med-base-8B	47	64.1	56.2	55.8

a Y-KNOT: Your-Knowledgeable Navigator of Treatment.

b KorMedMCQA dataset is freely available on Hugging Face [29]

### Automatic Drafting of Clinical Documents

For ED discharge summary, the AI agent drafts the whole contents in one paragraph, which includes past medical histories, reason for the visit, and the details of specialty consultations or treatments. In response to the urgent and fast-paced nature of the ED, the outputs are designed to be as concise as possible, meeting the specific requirements of the physicians.

For preanesthetic assessment, the agent drafts a patient’s background information required for preparing anesthesia, including basic information, past medical histories, medications, examination results, and other specialty consultation histories. Contents requiring medical judgment, such as anesthesiologist’s opinion or premedication guides, or American Society of Anesthesiologists (ASA) classification, were excluded as an LLM that makes medical judgements could be risky.

**Figure 3.**
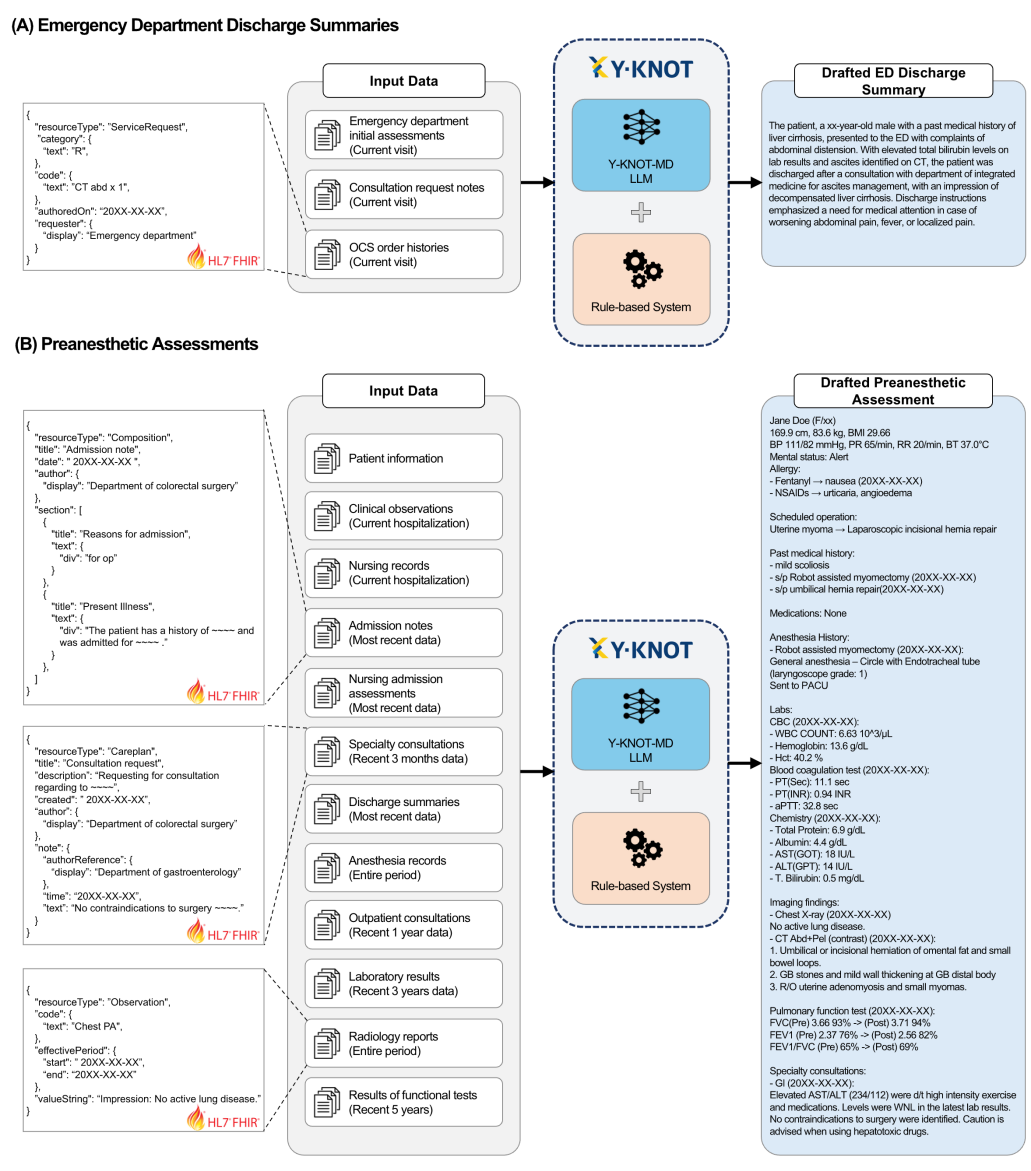
Examples of input data types and subsequent output contents of auto-generated drafts. All medical records used as input data are converted into Fast Healthcare Interoperability Resource (FHIR) standards. Criteria for selecting input data are stated in parentheses. Note that the examples provided in the figure are simplified versions of the actual data, which originally contains a mixture of Korean and English. The untranslated figure can be found in Multimedia Appendix 2. (A) An example of input data types and output contents of a drafted emergency department discharge summaries; (B) An example of input data types and output contents of drafted preanesthetic assessments. ED: emergency department; LLM: large language model; OCS: order communication system; Y-KNOT: Your-Knowledgeable Navigator of Treatment.

Detailed examples of generated drafts are provided in Figure 3.

### Integration and Implementation in Clinical Practice

**Figure 4.**
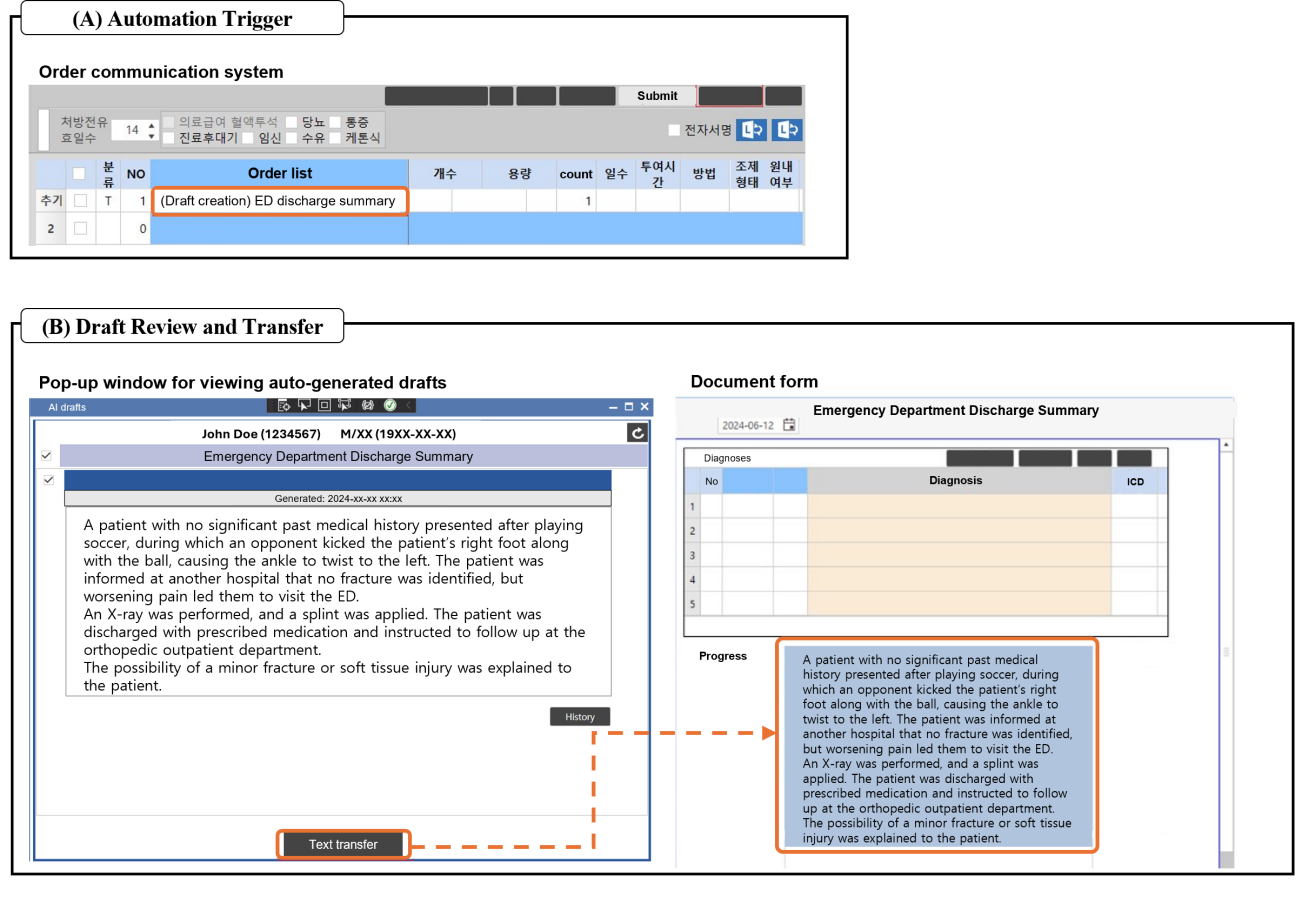
User interaction with the EHR system for automatic clinical drafting. (A) Requesting a draft creation through the order communication system; (B) Reviewing the auto-generated draft in the pop-up window. ED: emergency department.

The Y-KNOT service is currently deployed at Severance Hospital for real-world use. Since the agent is fully integrated into the EHR system, the drafting process is automatically triggered through two familiar physicians’ workflows. For ED discharge summaries, physicians can initiate drafting by placing a “draft creation” order, similar to medication orders, due to the need for prompt creation in acute care settings (Figure 4A). For preanesthetic assessments, which are associated with scheduled surgeries, the system generates drafts in batch according to a predetermined schedule. As a physician opens a form for documentation, auto-generated drafts show up in the pop-up window (Figure 4B). No external programs other than the EHR system are required to use the LLM. The entire process is similar to usual documentation workflows, except physicians can now load drafts with a single click instead of writing them from scratch. This also prevents potential risks of adversarial attacks [30] by keeping users away from instructing the LLM. Videos demonstrating the actual clinical use of the service are available in MultimediaAppendices 3  4.

**Figure 5.**
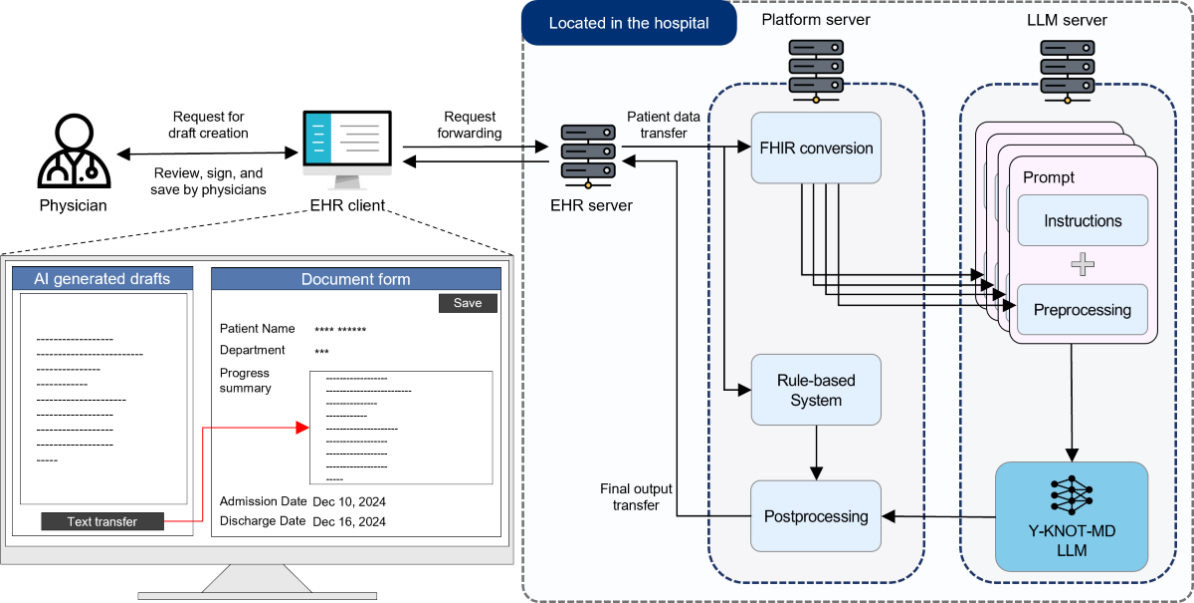
Overview of the automated drafting process with the AI agent in the EHR system. AI: artificial intelligence; EHR: electronic health record; FHIR: Fast Healthcare Interoperability Resource; LLM: large language model; Y-KNOT: Your-Knowledgeable Navigator of Treatment.

When the drafting is initiated, relevant patient records in FHIR format are transmitted from the EHR server to the Y-KNOT system, which processes them using a combination of LLM and rule-based approaches. The system preprocesses these standardized records into multiple prompts, each designed to extract specific aspects of the document. The LLM processes these prompts independently and generates outputs which are eventually synthesized into a comprehensive document draft. This final draft is returned to the EHR for physician review and approval. This automatic process (Figure 5) operates through predefined application programming interfaces (APIs) that specify data exchange formats between system components.

### Clinical Performance and Impact Assessment

The mean scores graded on drafted ED discharge summaries were 4.78 for consistency, 4.60 for coherence, 4.55 for fluency, 4.72 for relevance, 4.73 for safety, 3.95 for subjective satisfactory rate, and 3.32 for usability. The mean scores on drafted preanesthetic assessments were 3.29 for consistency, 3.86 for coherence, 4.23 for fluency, 3.37 for relevance, 3.88 for safety, 3.14 for subjective satisfactory rate, and 2.58 for usability. Additionally, out of 200 individual ratings on the impact on decision-making of preanesthetic assessments (2 raters evaluating 100 drafts), 69 (34.5%) were judged to be positive and 98 (49.0%) as having no impact, while 33 (16.5%) were judged to be negative (Table 4).

**Table 4.** Clinical evaluation results on drafts generated by the Y-KNOT^a^ AI agent.

Metrics	ED^b^ discharge summaries (n=200)	Preanesthetic assessments (n=200)
Consistency, mean (SD)	4.78 (0.56)	3.29 (1.10)
Coherence, mean (SD)	4.6 (0.75)	3.86 (0.82)
Fluency, mean (SD)	4.55 (0.73)	4.23 (0.69)
Relevance, mean (SD)	4.72 (0.61)	3.37 (0.91)
Safety, mean (SD)	4.73 (0.63)	3.88 (0.94)
Subjective satisfactory rate, mean (SD)	3.95 (1.03)	3.14 (1.10)
Usability^b^, mean (SD)	3.32 (0.76)	2.58 (0.87)
Impact on decision-making, n (%)		
Positive impacts	—	69 (34.5)
No impacts	—	98 (49.0)
Negative impacts	—	33 (16.5)

a Y-KNOT: Your-Knowledgeable Navigator of Treatment.

b ED: Emergency Department.

## Discussion

### Implications

The Y-KNOT project demonstrates a successful implementation of a bilingual on-premises LLM-based clinical drafting system that seamlessly integrates with existing EHR workflows in a high-throughput health care setting. Through close collaboration with stakeholders, we addressed several critical challenges.

Our decision to use a small model was crucial for real-world deployment, as larger models require substantial computational resources and costs. Although smaller models may have limitations in processing lengthy contexts and complex medical information, proper instruction-tuning enables them to perform specific tasks on par with larger models [31]. While initial clinical evaluation results of our model were modest, we prioritized rapid development using a small model to address the hospital’s pressing clinical needs. We transparently disclosed the evaluation results to all stakeholders and educated physicians prior to deployment regarding the possibility of errors in model outputs, with specific examples provided. After the deployment, discharge summary documentation completion rates in the Emergency Department improved from 92.7% in Apr-May 2024 to 98.0% in Apr-May 2025. Our experience demonstrates that carefully optimized smaller models can effectively support specific clinical drafting tasks when combined with thoughtful implementation strategies.

Moreover, our small model could address the unique challenges of resource-limited health care settings. South Korea’s health care system, while renowned for its accessibility, operates at significantly low costs, with the average cost per outpatient visit at tertiary hospitals being less than US $15, whereas in the United States, it exceeds US $100 [32]. This cost structure makes it financially unfeasible to deploy large-scale LLMs as the operational costs would significantly exceed the revenue per visit. Currently, the operational costs of the Y-KNOT service are solely covered by the hospital, but a national funding strategy could offer a more efficient approach for broader implementation in the future.

South Korea’s health care system is also highly efficient, with outpatient consultation times averaging merely 4.2 minutes [33], which is significantly shorter than the 20 minutes in the US [34]. This extreme time constraint presented both an opportunity and a challenge: while it highlighted an urgent need for documentation assistance, it also demanded exceptional efficiency in implementation. We addressed this challenge through strategic EHR integration, enabling documentation drafting to occur concurrently with other clinical tasks which eliminated perceived latency and maintained the rapid pace of clinical practice. This approach demonstrates how AI can be successfully integrated even in highly time-constrained, cost-sensitive clinical environments without disrupting established workflows.

To ensure scalable deployment across different health care institutions, we standardized all document templates to FHIR format and implemented API-based data exchange. As of December 2022, the Ministry of Health and Welfare in South Korea has established a taskforce to implement a 5-year strategy to accelerate health data standardization, which includes the specific task of developing and deploying Korea-specific FHIR standards [35]. In line with this initiative, we created a system that can be readily deployed to any EHR system that adheres to FHIR standards. This architectural decision not only ensures interoperability but also significantly reduces the technical barriers for other health care institutions wanting to implement similar AI-assisted documentation systems.

### Limitations

Our study has several limitations. First, clinical evaluation involved only 2 personnel per document type, potentially introducing bias due to small sample size. Second, we have not validated its performance across multiple institutions. Multicenter implementation studies would be crucial to establish the generalizability of our approach and identify potential institution-specific adaptation requirements. Third, this study does not include prospective results measuring the system’s impact on physician workload and documentation efficiency. Previous studies have raised concerns that the need for validating AI-generated outputs might paradoxically increase physician workload [36], making it crucial to evaluate the actual time savings through rigorous clinical studies [37,38]. We are actively conducting such prospective studies and plan to report our findings in future publications. Impacts on clinical decision-making or patient outcomes should also be assessed through long-term studies. Fourth, the financial implications remain to be fully understood. While there are expectations of cost benefits from AI implementation in health care [39], recent studies of similar technologies like ambient-listening AI have shown no significant financial advantages [40]. Future research should address these limitations through in-depth analyses with multicenter implementation studies, prospective evaluations of efficiency gains, clinical impact, and cost-effectiveness.

### Conclusions

This study provides a comprehensive account of developing and integrating an LLM-based AI agent for clinical drafting in routine clinical practice. We developed a specialized LLM by taking into consideration issues such as data sovereignty, bilingual challenges, and cost-effectiveness. In collaboration with various stakeholders, we integrated this solution with the EHR system to ensure practical usability by physicians without interruption of existing workflow.

## Supplementary material

10.2196/76848 Multimedia Appendix 1 Hyperparameter settings for model training.

10.2196/76848 Multimedia Appendix 2 Untranslated examples of input data types and subsequent output contents of auto-generated drafts.

10.2196/76848 Multimedia Appendix 3 Demonstration video for drafting emergency department discharge summaries using the Y-KNOT system.

10.2196/76848 Multimedia Appendix 4 Demonstration video for drafting preanesthetic assessments using the Y-KNOT system.

10.2196/76848 Checklist 1 i-CHECK-DH checklist.

## Abbreviations

AI: artificial intelligence
API: application programming interfaces
EHR: electronic health record
FHIR: Fast Healthcare Interoperability Resource
LLM: large language model
Y-KNOT: Your-Knowledgeable Navigator of Treatment
