# Supplementary material for: A Bilingual On-Premises AI Agent for Clinical Drafting: Implementation Report of Seamless Electronic Health Records Integration in the Y-KNOT Project
Source: JMIR Med Inform. 2025 Nov 24;13:e76848. doi: 10.2196/76848 (PMC12643392; doi:10.2196/76848)
Supplement: Multimedia Appendix 1 [file medinform-v13-e76848-s001.docx]

**Multimedia Appendix 2:** Hyperparameter settings for model training

| Model | Base model | Training method | Tokens | Hyperparameters | | | | | GPUs |
| --- | --- | --- | --- | --- | --- | --- | --- | --- | --- |
|  |  |  |  | Epochs | Batch size | Learning rate | Optimizer | DeepSpeed |  |
| Y-KNOT-med-base | Luxia2-8B | Pretraining | 32K | 1 | 128 | 1e-5 | Adam | ZeRO-3 | NVIDIA H100 80GB × 8 |
| Y-KNOT-MD | Y-KNOT-med-base | Supervised finetuning | 32K | 1 | 48 | 1e-6 | Adam | ZeRO-3 | NVIDIA H100 80GB × 8 |
