# Supplementary figures and images for: A Bilingual On-Premises AI Agent for Clinical Drafting: Implementation Report of Seamless Electronic Health Records Integration in the Y-KNOT Project
Source: JMIR Med Inform. 2025 Nov 24;13:e76848. doi: 10.2196/76848 (PMC12643392; doi:10.2196/76848)

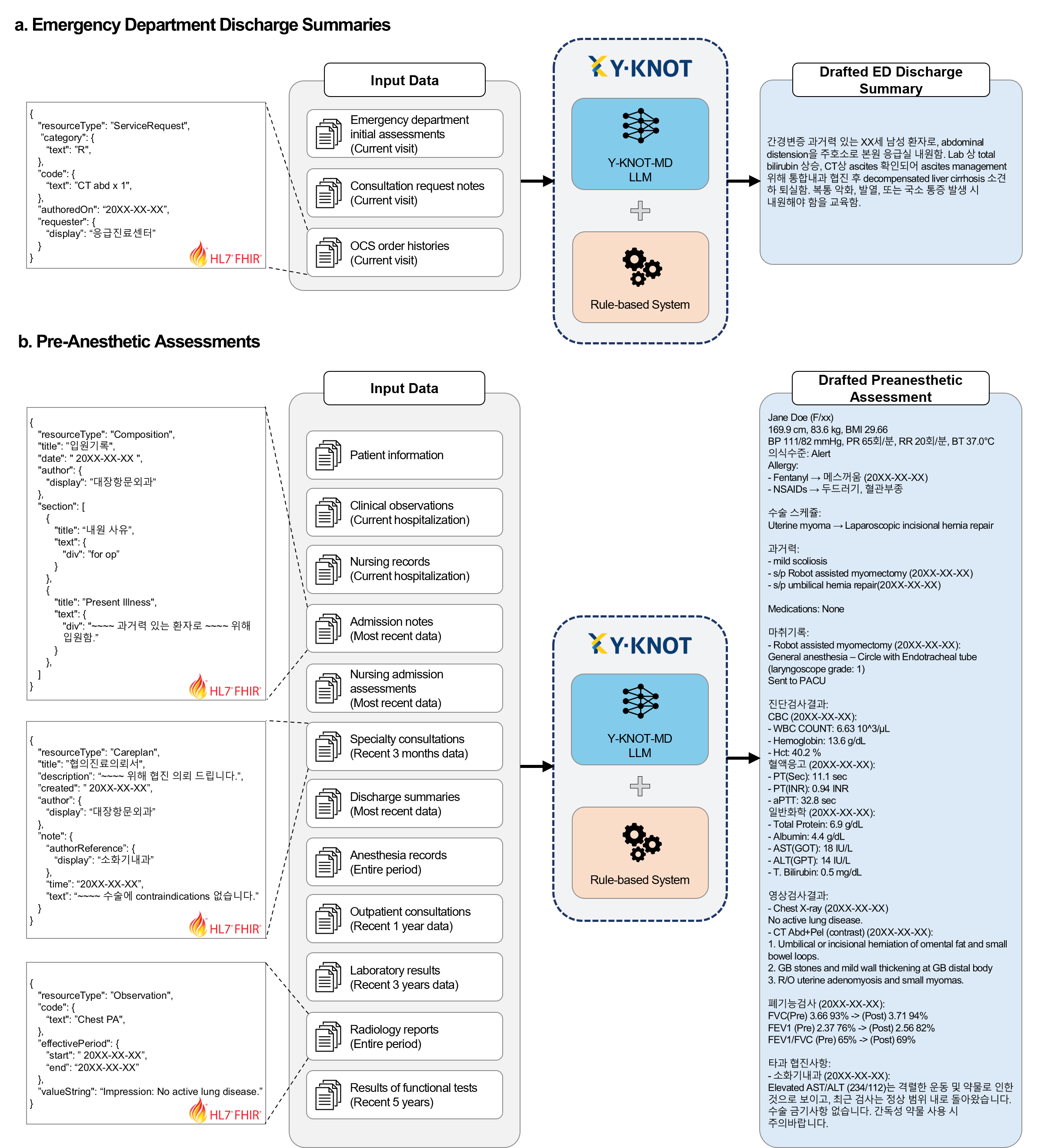

Supplement: Multimedia Appendix 2 [file medinform-v13-e76848-s002.png]
