# Supplementary material for: A Bilingual On-Premises AI Agent for Clinical Drafting: Implementation Report of Seamless Electronic Health Records Integration in the Y-KNOT Project
Source: JMIR Med Inform. 2025 Nov 24;13:e76848. doi: 10.2196/76848 (PMC12643392; doi:10.2196/76848)
Supplement: Checklist 1 [file medinform-v13-e76848-s005.docx]

**Multimedia Appendix 1.** Checklist of iCHECK-DH guidelines. iCHECK-DH: Guidelines and Checklist for the Reporting on Digital Health Implementations.

| section |  | Item | Description | RELEVANT TEXT |
| --- | --- | --- | --- | --- |
| Title | 1 | Title  (M^[[1]](#footnote-1)^) | Identification as an implementation report, and description of the implementation in the title and/or keywords | A Bilingual On-premise AI agent for Clinical Drafting: Implementation Report of Seamless EHR integration in the Y-KNOT Project |
| Abstract | 2 | Abstract  (M) | Provide a summary of the key elements of the implementation report, including a description of the implementation strategy, the intervention, defining the key elements of the implementation and health outcomes and specify the key KPIs/Outputs. We recommend describing the main aspects of the research in the following order: Background - Objectives - Methods - Implementation (Results) - Conclusions - (Optional: Trial Registration). | Background: Large Language Models (LLMs) have shown promise in reducing clinical documentation burden, yet their real-world implementation remains rare. Especially in South Korea, hospitals face several unique challenges such strict data sovereignty requirements and operating in environments where English is not the primary language for documentation. Therefore, we initiated the Your-Knowledgeable Navigator of Treatment (Y-KNOT) project, aimed at developing an on-premise bilingual LLM-based artificial intelligence (AI) agent system integrated with electronic health records (EHR) for automated clinical drafting.  Objective: We present Y-KNOT project and provide insights into implementing AI-assisted clinical drafting tools within constraints of healthcare system.  Methods: The project involved multiple stakeholders and encompassed three simultaneous processes: LLM development, clinical co-development, and EHR integration. We developed a foundation LLM by pretraining Llama3-8B with Korean and English medical corpora. During the clinical co-development phase, the LLM was instruction-tuned for specific documentation tasks through iterative cycles that aligned physicians’ clinical requirements, hospital data availability, documentation standards, and technical feasibility. The EHR integration phase focused on seamless AI agent incorporation into clinical workflows, involving document standardization, trigger points definition, and user interaction optimization.  Implementation (Results): The resulting system processes emergency department discharge summaries and preanesthetic assessments while maintaining existing clinical workflows. The drafting process is automatically triggered by specific events, such as scheduled batch jobs, with medical records automatically fed into the LLM as input. The agent is built on-premises, locating all the architecture inside the hospital.  Conclusions: The Y-KNOT project demonstrates the first seamless integration of an AI agent into an EHR system for clinical drafting. In collaboration with various clinical and administrative teams, we could promptly implement a LLM while addressing key challenges of data security, bilingual requirements, and workflow integration. Our experience highlights a practical and scalable approach to utilizing LLM-based AI agents for other healthcare institutions, paving the way for broader adoption of LLM-based solutions. |
| Introduction | 3 | Context  (M) | Describe the geographical areas, organizations, target populations and implementation context. Consider social, cultural, economic, political, health care and organizational barriers, infrastructures and facilitators that may influence implementation elsewhere. Explicitly highlight whether a national digital health strategy exists and whether implementation is aligned with the strategy.  Describe the stage of the implementation (Developing or Adapting Solution / Piloting and Evidence generation / Package and Advocacy /Acceleration / Deploying / Scaling up / Hand over or Complete).^[[2]](#footnote-2)^ | Large Language Models (LLMs) have recently garnered significant attention, raising expectations for their applications in healthcare systems, encompassing clinical care support, research, and education [1, 2]. However, most research has focused on implementations in the United States (US), and these solutions have yet to demonstrate meaningful reductions in administrative burden, as they mainly address tasks related to medical knowledge [3].  South Korea's healthcare system is renowned for its efficiency, offering low costs with high accessibility and quality. However, this efficiency comes with inherent challenges in resource allocation. Healthcare providers often manage substantial workloads, seeing many patients in limited time frames. This situation has been particularly exacerbated by recent mass resignation of residents, which has left tertiary hospitals facing a critical shortage of human resources [4, 5]. These circumstances underscore an urgent demand for meaningful assistance from LLMs.  Clinical documentation represents a significant burden for healthcare providers [6, 7], and there is growing optimism about LLMs' potential to alleviate this burden [8, 9]. Clinical documentation involves condensing previous records, a task LLMs excel at [10, 11].  Korea’s Ministry of Food and Drug Safety does not classify artificial intelligence (AI) software for documentation as a medical device unless it involves medical judgements [18], thereby exempting the requirement for regulatory approval. Nevertheless, these challenges hinder the widespread adoption of LLMs in Korea.  As of December 2022, the Ministry of Health and Welfare in South Korea has established a taskforce to implement a 5-year strategy to accelerate health data standardization, which includes the specific task of developing and deploying Korea-specific FHIR standards [35]. In line with this initiative, we created a system that can be readily deployed to any EHR system that adheres to FHIR standards |
|  | 4 | Problem statement  (M) | Description of the health care or public health problem, challenge, or deficiency that the implementation aims to address. (If applicable, include a reference to the 'health system challenge' of the WHO Classification of Digital Health Interventions^^[[3]](#footnote-3)^^ in the description) | However, implementing existing LLM solutions in South Korea faces several unique challenges. Korean medical regulations mandate that all medical records be stored exclusively on domestic servers or clouds [15], making it impossible to utilize foreign commercial services like ChatGPT [16]. Additionally, medical documents in Korea often exhibit mixed usage of Korean and English, requiring models capable of processing bilingual clinical notes effectively [17]. Korea’s Ministry of Food and Drug Safety does not classify artificial intelligence (AI) software for documentation as a medical device unless it involves medical judgements [18], thereby exempting the requirement for regulatory approval. Nevertheless, these challenges hinder the widespread adoption of LLMs in Korea.  Although some pilot projects have attempted incorporating LLMs within electronic health records (EHRs), full-scale integration in real clinical settings remains rare. Due to their separate interface, manually retrieving information from EHRs and typing it into LLMs may ironically be time-consuming. |
|  | 5 | Similar Interventions  (M) | Mention whether this implementation was inspired by another existing one, and if so, what is the added value of your intervention, if any, compared to the initial one? And what, if anything, has been done differently? | Accordingly, several studies have explored the capabilities of proprietary LLMs in generating clinical notes such as radiology referrals [12] or discharge summaries [13, 14].  Although some pilot projects have attempted incorporating LLMs within electronic health records (EHRs), full-scale integration in real clinical settings remains rare.  To address these challenges, we initiated the Your-Knowledgeable Navigator of Treatment (Y-KNOT) project, aimed at developing a hospital-dedicated AI agent that seamlessly integrates a small, bilingual LLM with existing systems for automatic clinical drafting. |
| Methods | 6 | Aims and Objectives  (M) | Describe the main objectives and the overall aim of the implementation. Describe how these will be measured using predefined primary and secondary outcome(s) and key performance indicators for this implementation and the expected intervention(s).  *For example: indicators or proxy-indicators measuring direct health outcomes (e.g., HbA1c for diabetic patients); Key Performance Indicators (e.g., number of users, number of users that are properly trained, user satisfaction); Indicator assessing a particular process (e.g., administrative time for patient admission);*  (If there was no evaluation, provide detailed explanation for reasoning) | We aim to demonstrate a practical approach to leveraging LLMs within the constraints of healthcare system, potentially offering a model for similar implementations in other limited-resource settings. The paper highlights the multidisciplinary process of the Y-KNOT project, key features of the final implementation, and presents a human evaluation of its feasibility. Our experience provides valuable insights into the challenges and opportunities of integrating AI-assisted clinical drafting tools in healthcare settings while maintaining compliance with local regulations and addressing specific linguistic requirements. |
|  | 7 | Blueprint summary  (M) | Describe the design and key features of the intervention and key points of the implementation strategy and roadmap. | The Y-KNOT project was conducted at Severance Hospital, a tertiary hospital in Seoul, South Korea. The project was initiated in June 2024 and the first service in routine clinical practice started in November 2024.  The project encompassed three major phases: medical foundation LLM development, clinical co-development, and EHR integration, which were carried out simultaneously. Figure 1 displays the overall project landscape. |
|  | 8 | Technical Design  (M) | Reasons for developing or choosing this tool. Does it combine several tools? Provide a brief description of the tool(s) (functionality and architecture) and how it fits into the health enterprise architecture and investment roadmap (if applicable). Indicate whether the solution is based on an existing solution or has been developed or purchased specifically for this intervention.  Describe the type of technology used (e.g., AI applications), license of the technology (open source, free, commercial, IP ownership etc.), include code documentation (if available), link to the application, link to wiki or project website. | We first developed ‘Y-KNOT-med-base’, a small, bilingual LLM for general medical purposes. We used Luxia 2 [21] developed by ‘Saltlux Inc.’ (Seoul, South Korea) as a base model, which was built upon Llama 3 (8 billion parameters) [22] and specialized for Korean through pretraining on 1.5 terabytes of general corpus datasets. We decided to utilize a small model for rapid project completion, minimal latency in clinical settings, and environmental and economic sustainability. To adapt the model for medical applications, we further trained it with 90 gigabytes (GB) of medical and 9 GB of general corpus datasets in Korean and English, consisting of open source and internally collected datasets. The pretraining data was augmented with instruction-response pairs for instruction pretraining [23], which enables better alignment with domain-specific tasks. The training was conducted outside of the hospital to ensure greater flexibility and broader reusability of the foundation model by other institutions. Hyperparameter settings are provided in Multimedia Appendix 2.  To assess its capability to understand medical knowledge, we evaluated ‘Y-KNOT-med-base’ on PubMedQA (biomedical question answering based on PubMed abstracts) [24] for English and KorMedMCQA (multi-choice question answering derived from licensing examinations for doctors, nurses, and pharmacists in South Korea) [25] for Korean. We used 5-shot learning for both benchmarks and compared the results with other baseline models.  Baselines for PubMedQA were selected from the state-of-the-art models on the PubMedQA leaderboard [27] whose parameter sizes were disclosed. . The accuracy scores of the models were taken from their original papers. Note that a leaderboard for KorMedMCQA does not exist, baseline models and their respective results were taken from the original KorMedMCQA paper, focusing specifically on non-proprietary multilingual models. Both PubmedQA and KorMedMCQA datasets are freely available on Hugging Face [28, 29].  To adapt the LLM for drafting specific document types – emergency department (ED) discharge summary and preanesthetic assessment – we instruction-tuned the Y-KNOT-med-base. We call the resulting model ‘Y-KNOT-MD’, which is an abbreviation for ‘Y-KNOT medical document’. Medical document data for the model prompts were selected from the hospital’s EHR database. Corresponding completions were prepared by physicians, addressing clinical needs while following the guidelines established by data scientists. The model was trained on 300 prompt-completion pairs for each document type. As the training involved patient data, it was conducted within the hospital environment to minimize the risk of data leakage. Details regarding hyperparameter settings are provided in Multimedia Appendix 2.  First, we screened medical document forms from the EHR system to be used for the actual service. Out of 2201 different document forms, total 989 forms were selected. The rest were excluded due to inconsistent usage, absence of textual content, or their association with surveys, referrals, palliative care or physical therapies. This decision was reached after numerous meetings with the medical records team and clinicians. Then, we standardized the selected forms based on Fast Healthcare Interoperability Resource (FHIR) [26] standards. This standardization not only enhanced interoperability for existing documentation but also established a robust framework for future development, ensuring long-term system scalability and maintainability.  Second, we mapped precise trigger points for AI agent activation to ensure assistance without disrupting existing clinical routines. The system supports both real-time triggers and batch processing. We carefully selected the optimal time for batch processing to minimize potential system load, and tested system latency to ensure that the integration would not impact the EHR's overall performance.  Third, we established a documentation display and a user interaction framework that maximized efficiency while preserving physician control over final documentation. The interface enabled quick review and editing of AI-generated content through intuitive controls for accepting, modifying, or rejecting suggestions. This design emphasized minimal click paths to streamline the documentation process. |
|  | 9 | Target  (M) | The target refers to the focus or recipient of the intervention. It is the specific person, group, system, or problem that the intervention aims to change or improve. The characteristics of the targeted "site(s)" (locations, staff, resources, etc.) for implementation and any eligibility criteria. The population targeted by the intervention and any eligibility criteria. | The Y-KNOT project was conducted at Severance Hospital, a tertiary hospital in Seoul, South Korea.  The Y-KNOT service is currently deployed at Severance Hospital for real-world use. |
|  | 10 | Data  (M) | Describe the data governance, including life cycle (collection, processing, storage, modification, sharing, suppression), the data ownership (mention whether patients actually have access to the data), data protection measures, confidential use of routine data, expected level of data integration, data for research, cross-border data agreement, if any, the applicable legal framework, and how the project complies with it. Data consent: Has patient consent been obtained? Describe the approach to data protection and cybersecurity (e.g. security by design, privacy by design, etc.) and where the data is hosted. (e.g., in-country, cloud based, hybrid model etc.). Describe, if applicable, the government preferences in terms of data policies. | All patient data used in this study were retrieved from the hospital’s research-purpose EHR database and deidentified prior to use, waiving the need for additional informed consent.  Medical document data for the model prompts were selected from the hospital’s EHR database. Corresponding completions were prepared by physicians, addressing clinical needs while following the guidelines established by data scientists  When the drafting is initiated, relevant patient records in FHIR format are transmitted from the EHR server to the Y-KNOT system, which processes them using a combination of LLM and rule-based approaches.  To ensure data sovereignty, all infrastructures including servers and databases were hosted within the hospital's secure on-premise environment. |
|  | 11 | Interoperability  (M) | Describe the interfaces (what other systems does the tool connect to) and the standards that were used (which specific ones and rationale of choice) (e.g., semantic ontologies such ICD as SNOMED, LOINC or technical standards such as HL7 FHIR, etc.). | Then, we standardized the selected forms based on Fast Healthcare Interoperability Resource (FHIR) [26] standards. This standardization not only enhanced interoperability for existing documentation but also established a robust framework for future development, ensuring long-term system scalability and maintainability.  To ensure scalable deployment across different healthcare institutions, we standardized all document templates to FHIR format and implemented API-based data exchange. As of December 2022, the Ministry of Health and Welfare in South Korea has established a taskforce to implement a 5-year strategy to accelerate health data standardization, which includes the specific task of developing and deploying Korea-specific FHIR standards [35]. In line with this initiative, we created a system that can be readily deployed to any EHR system that adheres to FHIR standards. |
|  | 12 | Participating entities  (M) | Describe the implementing organization(s): Type of organisation(s), mission, leadership, vision, etc.  Government involvement: Describe whether the government was involved in the implementation, at what level and at what stage(s).  Partners: Describe all partners (organisations) and their role in the implementation.  Funders: List all actors and stakeholders who have funded or invested in the development of the implementation (if different from the implementation, e.g. using an existing digital health intervention). Indicate their level of involvement in terms of funding.  Mention which entity will own the final product and intellectual property after the implementation phase. | The final LLM model developed in this project is jointly owned by Severance Hospital and ‘PHI Digital Healthcare Co., Ltd.’ (Seoul, South Korea).  Figure 1  The Y-KNOT project involved intensive collaboration with related departments, including physicians, data scientists, software engineers, and medical record specialists.  Corresponding completions were prepared by physicians, addressing clinical needs while following the guidelines established by data scientists. |
|  | 13 | Budget Planning  (M) | Describe the planned budget for implementation (include costs such as change management, user training, project management, technology pricing, total cost of ownership). If possible, include actual costs, otherwise describe the range or percentage of the total budget. Indicate the period covered by the budget. Describe the budget for the intervention (e.g. development, purchase or adaptation of a free tool); if possible include real costs, otherwise describe as a percentage of the total budget. Indicate the duration covered by the budget. | The total cost of the project, including all expenses such as equipment and labor, did not exceed US $1,500,000. |
|  | 14 | Sustainability  (M) | Describe the Business model including the sustainability model (financial, economic, environment etc.). If possible, put outcomes in relation to cost to assess sustainability. Describe long term exit strategies, and all dimensions considered to sustain the project after the end of funding. If applicable, describe potential institutionalization of the project. | Currently, the operational costs of the Y-KNOT service are solely covered by the hospital, but a national funding strategy could offer a more efficient approach for broader implementation in the future. |
| RESULTS | 15 | Coverage  (M) | Describe whether the coverage of implementation is international, national, regional or at the level of e.g. municipalities. If coverage is sub-national, describe the regions. Provide information on the relative importance of the coverage (e.g. % of eligible population covered). | The Y-KNOT service is currently deployed at Severance Hospital for real-world use. |
|  | 16 | Outcomes  (M) | Primary and other outcome(s) of the implementation. Detail the actual outcomes, using the pre-defined outcome measures (if applicable). | The ‘Y-KNOT-med-base’ achieved an accuracy score of 75.2 on the PubMedQA. Despite its relatively small size and absence of fine-tuning process, the performance was comparable to state-of-the-art baselines which were fine-tuned on larger parameter scales. The average accuracy score was 55.8 on the KorMedMCQA (doctor: 47.0, nurse: 64.1, pharmacist: 56.2), outperforming other multilingual pretrained models on all three exam categories. Detailed performance results are provided in Table 2.  The mean scores graded on drafted ED discharge summaries were 4.78 for consistency, 4.60 for coherence, 4.55 for fluency, 4.72 for relevance, 4.73 for safety, 3.95 for subjective satisfactory rate, and 3.32 for usability. The mean scores on drafted preanesthetic assessments were 3.29 for consistency, 3.86 for coherence, 4.23 for fluency, 3.37 for relevance, 3.88 for safety, 3.14 for subjective satisfactory rate, and 2.58 for usability. Additionally, out of 200 individual ratings on the impact on decision-making of preanesthetic assessments (2 raters evaluating 100 drafts), 69 (34.5%) were judged to be positive and 98 (49.0%) as having no impact, while (16.5%) were judged to be negative (Figure 6). |
|  |  | Lessons learned  (M) | Describe any lessons learned from the implementation experience that could be used to improve future outcomes. This could include, but is not limited to, success factors, implementation challenges or budget considerations.  Success factors: Describe factors that positively influenced the implementation (e.g. involvement of key stakeholders). Also describe contextual factors that may have positively influenced the results (e.g. new legal requirements that facilitated adoption).  Challenges to implementation: Describe challenges (process-related, such as resistance to change, but also technical). Include contextual factors that may have affected the achievement of outcomes such as an unexpected change of government, or 'opposing key players' who, despite potential participation, may hinder implementation (e.g. software companies managing regional digital health may act as barriers to innovation).  Budget: Describe whether the implementation budget was adhered to, and if not, why not. Also detail the expected operational costs (e.g. licence, maintenance, human resources, updates to in-house developments) to estimate the total cost of ownership. Include real costs, otherwise describe them as a percentage of the total budget.  What recommendations can be drawn from the lessons learned? | Our decision to utilize a small model was crucial for real-world deployment, as larger models require substantial computational resources and costs. Although smaller models may have limitations in processing lengthy contexts and complex medical information, proper instruction-tuning enables them to perform specific tasks on par with larger models [31]. While initial clinical evaluation results of our model were modest, we prioritized rapid development using a small model to address the hospital’s pressing clinical needs. We transparently disclosed the evaluation results to all stakeholders and educated physicians prior to deployment regarding the possibility of errors in model outputs, with specific examples provided. After the deployment, discharge summary documentation completion rates in the Emergency Department improved from 92.7% in Apr-May 2024 to 98.0% in Apr-May 2025. Our experience demonstrates that carefully optimized smaller models can effectively support specific clinical drafting tasks when combined with thoughtful implementation strategies.  Moreover, our small model could address the unique challenges of resource-limited healthcare settings. South Korea's healthcare system, while renowned for its accessibility, operates at significantly low costs, with the average cost per outpatient visit at tertiary hospitals being less than US $15, whereas in the US, it exceeds US $100 [32]. This cost structure makes it financially unfeasible to deploy large-scale LLMs as the operational costs would significantly exceed the revenue per visit. Currently, the operational costs of the Y-KNOT service are solely covered by the hospital, but a national funding strategy could offer a more efficient approach for broader implementation in the future.  South Korea's healthcare system is also highly efficient, with outpatient consultation times averaging merely 4.2 minutes [33], which is significantly shorter than the 20 minutes in the US [34]. This extreme time constraint presented both an opportunity and a challenge: while it highlighted an urgent need for documentation assistance, it also demanded exceptional efficiency in implementation. We addressed this challenge through strategic EHR integration, enabling documentation drafting to occur concurrently with other clinical tasks which eliminated perceived latency and maintained the rapid pace of clinical practice. This approach demonstrates how AI can be successfully integrated even in highly time-constrained, cost-sensitive clinical environments without disrupting established workflows.  To ensure scalable deployment across different healthcare institutions, we standardized all document templates to FHIR format and implemented API-based data exchange. As of December 2022, the Ministry of Health and Welfare in South Korea has established a taskforce to implement a 5-year strategy to accelerate health data standardization, which includes the specific task of developing and deploying Korea-specific FHIR standards [35]. In line with this initiative, we created a system that can be readily deployed to any EHR system that adheres to FHIR standards. This architectural decision not only ensures interoperability but also significantly reduces the technical barriers for other healthcare institutions wanting to implement similar AI-assisted documentation systems. |
|  | 17 |  |  |  |
|  | 18 | Unintended consequences  (NM^[[4]](#footnote-4)^) | Describe unintended consequences (positive or negative), harms or negative side-effects (if any). | - |
| Discussion | 19 | Conclusion  (M) | Summary of the conclusions and future implications. | This study provides a comprehensive account of developing and integrating an LLM-based AI agent for clinical drafting in routine clinical practice. We developed a specialized LLM by taking into consideration issues such as data sovereignty, bilingual challenges, and cost-effectiveness. In collaboration with various stakeholders, we integrated this solution with the EHR system to ensure practical usability by physicians without interruption of existing workflow. |
| General | 20 | General  (NM) | If applicable, include statement(s) on regulatory approvals (including, as appropriate, ethical approval, governance approval), trial or study registration (availability of protocol), and conflicts of interest. For implementation reports with a research component, ethical approval or a waiver from an appropriate ethics committee is required. For those without a research component, ethical considerations may still be relevant, but do not necessarily require approval or waiver. Authors may consult [this article](https://implementationscience.biomedcentral.com/articles/10.1186/1748-5908-6-32)^[[5]](#footnote-5)^ for further guidance on ethical considerations in their specific context" | This study was reviewed and approved by the Institutional Review Board (IRB No. 4-2023-003) and the Data Review Board (DRB No. 24-01-005) of Severance Hospital. All patient data used in this study were retrieved from the hospital’s research-purpose EHR database and deidentified prior to use, waiving the need for additional informed consent.  This research was supported by PHI Digital Healthcare and is associated with Patent Applications PATENT-2025-0039190, PATENT-2025-0039191, PATENT-2025-0039192, PATENT-2025-0039193, and PATENT-2025-0039194. SCY reports grants from Daiichi Sankyo. He is a coinventor of granted Korea Patent DP-2023-1223 and DP-2023-0920, and pending Patent Applications DP-2024-0909, DP-2024-0908, DP-2022-1658, DP-2022-1478, and DP-2022-1365 unrelated to current work. SCY is a chief executive officer of PHI Digital Healthcare. HK was an employee of PHI Digital Healthcare during this study. SYL is an employee of PHI Digital Healthcare. JEK, STK, and DRK are employees of Saltlux Inc. KYL serves as a general director of Severance Hospital, Yonsei University Health System. Other authors have no potential conflicts of interest to disclose. |

1. M: Mandatory item [↑](#footnote-ref-1)
2. *Stages adapted from WHO Digital Health Atlas* [↑](#footnote-ref-2)
3. <https://apps.who.int/iris/bitstream/handle/10665/260480/WHO-RHR-18.06-eng.pdf?sequence=1&isAllowed=y> [↑](#footnote-ref-3)
4. NM : Non-mandatory item [↑](#footnote-ref-4)
5. Eccles, M. P., Weijer, C., & Mittman, B. (2011). Requirements for ethics committee review for studies submitted to Implementation Science. Implementation science, 6(1), 1-3. [↑](#footnote-ref-5)
